# Fermented Apple Juice Reduces the Susceptibility of Offspring Mice to Food Allergy Exacerbated by Maternal High-Fat Diet

**DOI:** 10.3390/nu17111927

**Published:** 2025-06-04

**Authors:** Jing Ma, Jian Yu, Yining Jia, Zining Luo, Xin Yang, Huzhong Li, Fangyu Long

**Affiliations:** 1 College of Food Science and Engineering, Northwest A&F University, Xianyang 712100, China; 2 College of Animal Science and Technology, Northwest A&F University, Xianyang 712100, China; 3 NHC Key Laboratory of Food Safety Risk Assessment, China National Center for Food Safety Risk Assessment, Beijing 100022, China

**Keywords:** maternal diet, offspring, high-fat diet, allergy, gut microbiota, acylcarnitine

## Abstract

**Background:** Food allergy (FA) is associated with dietary habits, antibiotic use, living environment, and delivery method. Pregnancy and lactation represent critical periods for neonatal immune system development. **Methods**: This study investigated the relationship between maternal dietary habits and FA risk in offspring. Pregnant C57BL/6J mice (8-week-old males and females) were fed either a high-fat diet (HFD) or HFD supplemented with fermented apple juice (FAJ) during pregnancy and lactation. Offspring were nursed by their respective dams until weaning at 21 days postpartum, followed by ovalbumin (OVA) sensitization. Lipid profiles, acylcarnitines, immunological, and histopathological analyses were performed. Gut microbiota composition and serum markers were also assessed. **Results**: The findings indicated that maternal HFD had a negative impact on OVA-sensitized offspring mice. Early-life FAJ intervention modulated gut microbiota alterations and alleviated maternal HFD-worsened allergic symptoms through Th1/Th2 and Th17/Treg immunity balance and intestinal barrier repair. Maternal serum triglyceride and total cholesterol levels, along with gut microbiota profiles, significantly influenced offspring gut microbiota composition. Moreover, reduced short-chain and medium-chain acylcarnitines in offspring may be associated with increased allergy risk. **Conclusions**: Maternal HFD during pregnancy and lactation disrupted gut microbiota balance and exacerbated offspring FA susceptibility. These findings provide a scientific foundation for developing early-life FA prevention strategies.

## 1. Introduction

Allergic diseases mainly include atopic dermatitis, food allergy (FA), asthma, allergic rhinitis, etc., with a higher prevalence observed in children. FA is an IgE-mediated adverse reaction that occurs when a sensitized individual is re-exposed to the same food allergen [1]. FA affects approximately 3–4% of adults and 6–8% of children [2]. Emerging evidence suggests that food allergy (FA) development is a multifactorial process involving dietary factors, environmental influences, genetic susceptibility, epigenetic regulation, and perinatal determinants [3]. Considering the complex interplay of factors in the pathogenesis of FA, current evidence suggests that allergen avoidance is the most reliable method of preventing FA [4]. Pregnancy and lactation are critical periods for the development of the neonatal immune system, and environmental factors could disrupt immune programming early in life, potentially increasing the risk of FA development and reducing the host’s resistance to diseases [5]. The dietary adiposity hypothesis proposed by Gideon Lack suggests a connection between FA, obesity, and metabolic diseases, which may contribute to the rising prevalence of conditions such as diabetes and metabolic syndrome [6]. Maternal obesity has been associated with immune dysregulation in human neonates and could lead to intestinal inflammation and increased intestinal permeability in offspring, thereby promoting susceptibility to FA [7,8]. Additionally, a previous cohort study involving Chinese twins confirmed that maternal dyslipidemia may elevate the risk of FA in offspring [9].

Maternal nutrition plays a crucial role in influencing the risk of immune-mediated disorders in offspring through epigenetic functions [10]. Thorburn et al. [11] demonstrated that a high-fiber diet during pregnancy reduced the incidence of allergic diseases in children by increasing the number of regulatory T cells and positively influencing gut microbiota. Additionally, maternal probiotic supplementation during pregnancy and early infancy (0–6 months) may confer protective effects against the development of FA in offspring. Notably, the use of oral *Lactobacillus rhamnosus* during pregnancy was found to decrease the occurrence of FA in infants with a family history of allergic sensitization at 2–4 months of age [12]. Furthermore, Zhao et al. [13] discovered that supplementing female rats with *Lactobacillus rohitaensis* during gestation could prevent the onset of FA by regulating the balance between Th1 and Th2 immune responses, increasing the proportion of Treg cells, and altering the gut microbiota in the offspring.

Currently, the management of FA primarily involves avoiding allergens and having emergency medications available for accidental ingestion. Therefore, it is crucial to identify modifiable factors in early life to intervene in FA as early as possible. Functional foods and bioactive natural compounds may serve as effective preventive interventions for FA, demonstrating favorable safety profiles, practical administration, and cost-effectiveness [14]. In the late 1920s, Middleton [15] highlighted the anti-allergic effects of citrus, marking the first report on the anti-allergic properties of foods with natural ingredients. The research provided a new avenue for exploring the diverse functions of foods and potential preventive measures against FA. Dietary interventions have a rich history in the prevention of FA, and the variety of available diets highlights the potential for multiple approaches to preventing and treating FA.

The mechanisms underlying how early-life HFD exposure affects susceptibility to FA in offspring remain poorly understood. This study evaluated the impact of maternal HFD during pregnancy and lactation on offspring FA risk development. In addition, it is crucial to emphasize the importance of maternal nutritional supplementation during pregnancy and lactation. A comprehensive investigation of both the mechanisms through which maternal diet influences offspring FA risk development and the identification of factors affecting offspring FA susceptibility is essential for developing effective preventive and therapeutic strategies against FA.

## 2. Materials and Methods

### 2.1. Experimental Animal Design

**Figure 1:**
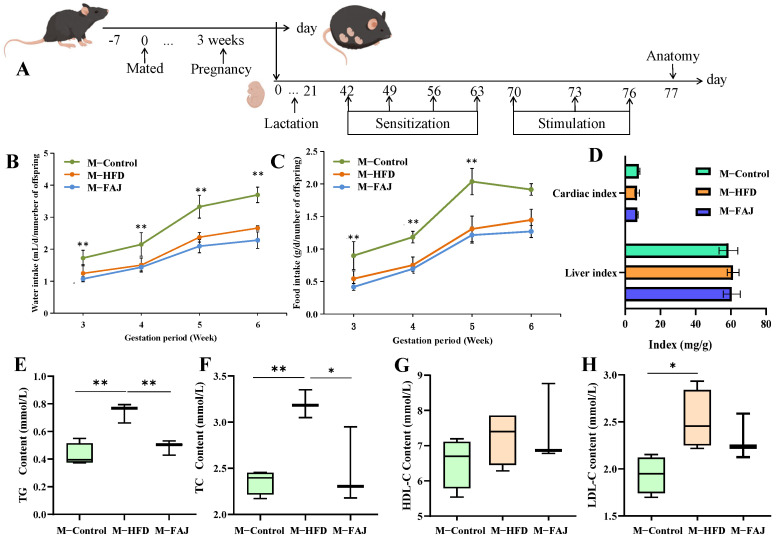
Processing timeline (**A**). Maternal water intake (**B**), maternal food intake (**C**), and organ index (**D**) during pregnancy and lactation. The content of TG (**E**), TC (**F**), HDL-C (**G**), and LDL-C (**H**) in maternal mice with different diets during pregnancy and lactation. Each value is expressed as the mean ± SD (*n* = 6). ** *p* < 0.01; * *p* < 0.05.

Male and female C57BL/6J mice (8 weeks old) were procured from SPF Biotechnology Co., Ltd. (Beijing, China). All animal procedures were approved by the Animal Care Committee of Northwest A&F University (XN2023-1206) and conducted in strict accordance with the China Council on Animal Care guidelines. The female mice were mated with male mice (housing one male with two females for 24 h), then the pregnant female mice were housed individually. Following parturition, the pups remained with their biological dams under standard nursing conditions until weaning at 21 days of age. During the lactation period, each dam was housed separately in standard cages with free access to food and water (Figure 1A). The animals were allocated into three groups: Control group (M-Control): The maternal mice were fed with a standard diet (SD) (*n* = 6). Model group (M-HFD): The maternal mice were fed with a high-fat diet (HFD, 60% fat, XTHF60-1, Jiangsu Xietong Pharmaceutical Bio-engineering Co., Ltd., Nanjing, China) (n = 6). The pregnant mice were randomly divided into three groups: Experiment group (M-FAJ): the maternal mice were fed with HFD and FAJ (0.15 mL/10 g) during pregnancy and lactation (*n* = 6) [16], and fermented apple juice was prepared according to our previous methods [17]. The offspring mice in the M-Control, M-HFD, and M-FAJ groups were divided into different groups: offspring mice in the M-Control group (O-M-Control) (male = 6; femal = 5), offspring mice in the M-HFD group (O-M-HFD) (male = 8; female = 7), offspring mice in the M-HFD group and sensitized with ovalbumin (OVA, SDS-PAGE ≥ 60%, Beijing, China) (O-M-HFD-A) (male = 8; female = 7), and offspring mice in the M-FAJ group and sensitized with OVA (O-M-FAJ-A). The mice were sensitized orally on days 0, 7, 14, and 21 with OVA (1.0 mg) and cholera toxin (10 μg) (Solarbio, Beijing, China). followed by challenging with 50 mg OVA on days 28, 31, and 34. All of the offspring mice were fed with SD. Mice with obvious allergic symptoms were selected to continue the subsequent experiments. All mice were anesthetized with isoflurane after the last OVA challenge, then sacrificed after serum collection. The cecal contents and jejunum tissues were stored at −80 °C for further analysis.

### 2.2. Biochemical Analysis

Serum samples were collected and analyzed for lipid profiles, including triglycerides (TG), total cholesterol (TC), low-density lipoprotein cholesterol (LDL-C), and high-density lipoprotein cholesterol (HDL-C), using commercially available assay kits (Nanjing Jiancheng Bioengineering Institute, Nanjing, China) following the manufacturer’s protocols.

### 2.3. Determination of Apparent Indexes

Spleen, thymus, cardiac, and liver indexes were calculated. Weekly food intake and water intake were recorded. Body temperature was recorded by anal thermometer (Hongou Chengyun, Beijing, China) and infrared thermography (LAT Science system with Testo IRSoft analysis, Beijing, China).

### 2.4. Histopathological Observation

Jejunum (0.5 cm) from mice was fixed with 4% formaldehyde for 24 h, then sectioned at 4–5 μm and stained with hematoxylin and eosin (HE). Finally, a fluorescent inverted microscope (HT7700, Hitachi Limited, Tokyo, Japan) was used to observe the morphological structure of the jejunum tissues.

### 2.5. Detection of Serum Indicators

ELISA kits were utilized to estimate the levels of IgE, IgG1, IgG2a, IL-4, IL-17A, TNF-*α*, IL-6, IL-10, and IFN-*γ* in the mice serum according to the manufacturer’s instructions (Ruixin, Quanzhou, China).

### 2.6. Gut Microbiota Analysis

Colon tissue samples were collected and immediately snap-frozen in liquid nitrogen for subsequent 16S rRNA gene sequencing and liquid chromatography-tandem mass spectrometry (LC-MS/MS) analyses. Comprehensive protocols for sample preparation, chromatographic separation, and mass spectrometric detection parameters have been previously described [18].

### 2.7. Determination of Acylcarnitine Content in Serum

A 50 μL sample was placed in a 1.5 mL centrifuge tube, then 150 μL of methanol, which contained C0-D3 (2000 ng/mL), C2-D3 (2000 ng/mL), C4-D3 (200 ng/mL), C8-D3 (200 ng/mL), and C16-D3 200 (ng/mL) internal standards (PR-31200, Cambridge Isotope Laboratories, Tewksbury, Massachusetts, USA) was added. The vortex was shaken for 10 min and then centrifuged at 12,000 rpm for 10 min (4 °C). The supernatant (80 μL) was pipetted into a 250 μL cannula (VDAP-4025-6297E-100, ANPEL Laboratory Technologies, Shanghai, China). The separation was performed on an ACQUITY UPLC I-CLASS ultra-high-performance liquid chromatography column equipped with a Waters UPLC HSS T3 chromatographic column (1.7 μm, 2.1 mm × 150 mm, Waters, Milford, MA, USA) at a flow rate of 0.26 mL/min (50 °C). The mobile phase consisted of 0.1% formic acid in water (solvent A) and acetonitrile (solvent B). The gradient elution procedure of phase B was 0.0–3.5 min, 2%; 3.5–5.0 min, 50%; 5.0–7.0 min, 100%; and 7.0–9.0 min, 2%. A Waters XEVO TQ-S tandem quadrupole mass spectrometer system (XEVOTQ-S-Micro, Waters, MA, USA) was used for mass spectrometry analysis. Ion source temperature: 150 °C; positive ion source voltage: 3.0 kV; desolvation temperature: 500 °C; cone-well voltage: 30 V; cone-well gas flow rate: 10 L/h; desolvation gas flow rate: 950 L/h. Peak areas were calculated using Target Lynx software (v4.1) with an allowable error of 15 s in retention time, and quantitative results were obtained by single-site isotope internal standardization.

### 2.8. Statistical Analysis

All values are expressed as the mean ± standard deviation (SD). Normal distribution was examined by the Shapiro–Wilk test. Multiple treatment groups were compared using one-way ANOVA followed by the Tukey test or the non-parametric Kruskal–Wallis test. A value of *p* < 0.05 was considered statistically significant, and was considered significant differences. The gut microbiota and metabolites were analyzed on the online platform Majorbio Cloud Platform (www.majorbio.com (accessed on 20 June 2023)). The correlation diagrams are mapped using ChiPlot (https://www.chiplot.online/gene_cluster.html (accessed on 20 June 2023)). And the graphical abstract is drawn by Figdraw.

## 3. Results and Discussion

### 3.1. Effects of HFD on Epigenetic Indicators of Maternal Mice

The apple juice was pretreated by high hydrostatic pressure (HHP, 200 MPa) for 10 min and then fermented by *Lactobacillus plantarum PA01* for 24 h. The FAJ contained total phenols at 192.6 mg/L and achieved a viable cell count of 7.58 log CFU/mL [17]. Comparative analysis revealed that the 200 MPa + Fermentation group showed significant metabolic changes relative to untreated, 200 MPa, and pasteurized + fermentation apple juice. Notably, the levels of L-Iditol, Osajin, D-Raffinose, and L-phenylalanine were significantly increased in the 200 MPa + Fermentation group (Figure S1), which may be potential substances for the biological activity of HHP-pretreated FAJ. During pregnancy and lactation, it was observed that there was a gradual increase in food intake and water intake. However, 6 weeks of HFD intervention led to reduced consumption (Figure 1B,C), while liver and cardiac indexes remained unchanged (Figure 1D). As shown in Figure 1E–H, TC (*p* = 0.0078), TG (*p* = 0.0018), and LDL-C (*p* = 0.0308) levels in serum were significantly increased by 35.71%, 173.19%, and 44.95% in the M-HFD group compared with the M-Control group, respectively. This result suggested that maternal HFD in pregnancy and lactation leads to dyslipidemia in mothers. After the FAJ intervention in maternal mice fed with HFD during pregnancy and lactation, there were significant decreases in TC (*p* = 0.0237) and TG (*p* = 0.0084), indicating that FAJ had a certain ameliorating effect on dyslipidemia. Studies have been conducted to show that fermented products can regulate dyslipidemia caused by HFD. Boby et al. [19] indicated that fermented Pyrus ussuriensis Maxim extract greatly improved obesity-related biomarkers, including TC, leptin, active ghrelin, etc., in rats. Cloudy apple juice fermented by *Lactobacillus* could regulate blood lipid levels and prevent obesity [16].

### 3.2. Impacts of HFD on Gut Microbiota of Maternal Mice

**Figure 2:**
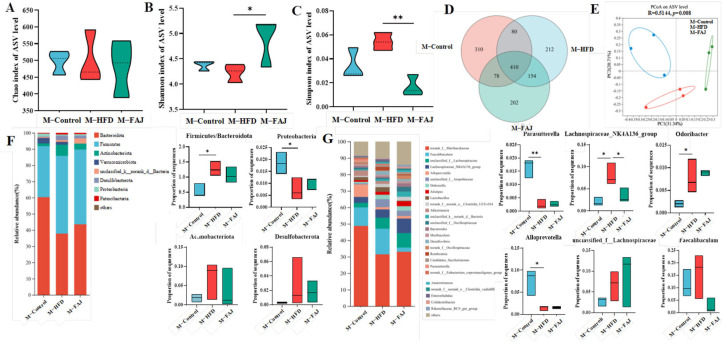
Effect of FAJ intervention on the richness and diversity of gut microbiota in maternal mice on an HFD during pregnancy and lactation. (**A**) Chao index. (**B**) Shannon index. (**C**) Simpson index. (**D**) Veen. (**E**) PCoA scores plot of gut microbiota at the ASV level. (**F**) The composition of gut microbiota at the phylum level. (**G**) The composition of gut microbiota at the genus level. Each value is expressed as the mean ± SD (*n* = 3). * *p* < 0.05 and ** *p* < 0.01.

The gut microbiota of mice was analyzed using 16S rDNA amplicon sequencing. Alpha diversity, including richness and evenness, was assessed using Chao, Shannon, and Simpson indexes. FAJ intervention-induced changes in bacterial alpha diversity are shown in Figure 2A–C. In comparison to the M-Control group, the M-HFD group showed an increased Simpson index and reduced Shannon index (*p* > 0.05), both of which were modulated by FAJ intervention. The findings indicated that different maternal diets during pregnancy and lactation may affect gut microbiota richness and diversity. At the ASV level, the number of unique and shared bacteria is presented in Figure 2D. The M-Control group contained 310 kinds of unique bacteria, compared with 212 in the M-HFD group and 202 in the M-FAJ group, indicating that both HFD and FAJ interventions during pregnancy and lactation may alter gut microbiota composition. Principal coordinate analysis (PCoA) revealed community structural relationships in a reduced-dimensional space, with the closer the positional distribution, the more similar the composition. PCoA of beta diversity revealed significant compositional alterations in gut microbiota following HFD and FAJ interventions (Figure 2E) (*p* = 0.008). The clusters in the M-Control and the M-HFD groups were easily distinguished, and the M-FAJ group was separated from the M-HFD group, which means that dietary supplementation with FAJ significantly modulated gut microbial community richness.

A total of 40 genera and eight phyla were identified by species composition analysis, among which Bacteroidota, Firmicutes, Actinobacteriota, Verrucomicrobiota, Desulfobacterota, Proteobacteria, and Patescibacteria were the main phyla (Figure 2F). The Firmicutes/Bacteroidota ratio, which is associated with immune diseases [20], significantly increased in the M-HFD group (*p* = 0.0236) but decreased following FAJ intervention. Compared to the M-Control group, the M-HFD group showed reduced Proteobacteria abundance. Dietary interventions had no significant effect on Actinobacteriota and Desulfobacterota abundances. At the genus level, *norank_f__Muribaculaceae*, *Faecalibaculum*, *unclassified_f__Lachnospiraceae*, *Lachnospiraceae_NK4A136_group*, and *Alloprevotella* were the highest abundances (Figure 2G). *Odoribacter*, known for producing short-chain fatty acids that alleviate ulcerative colitis symptoms [21], and *Lachnospiraceae_NK4A136_group*, which contributes to acetic and butyric acid production, were significantly affected. *Odoribacter* has also been recognized as a gut strain positively associated with obesity and ulcerative colitis [22]. The results showed that maternal HFD during pregnancy and lactation increased the abundance of *Odoribacter* and decreased the proportions of *Parasutterella* and *Alloprevotella* in maternal mice. In addition, the *Lachnospiraceae_NK4A136_group* increased in the M-HFD group in comparison to the M-Control group and decreased to 8.06% in the M-FAJ group (*p* = 0.0406). FAJ intervention partially restored HFD-induced gut microbiota alterations. In addition, the abundances of *unclassified_f_Lachnospiraceae* and *Faecalibaculum* were decreased after FAJ intervention, which has been shown to have strong positive correlations with inflammation and obesity, impairing glucose and lipids leading to metabolic disorders [23,24]. These results illustrated that maternal HFD during pregnancy and lactation regulates gut microbiota composition, while FAJ intervention shows potential to promote beneficial flora and inhibit harmful microbiota.

### 3.3. Maternal HFD Influenced Epigenetic Indicators in Offspring Mice

**Figure 3:**
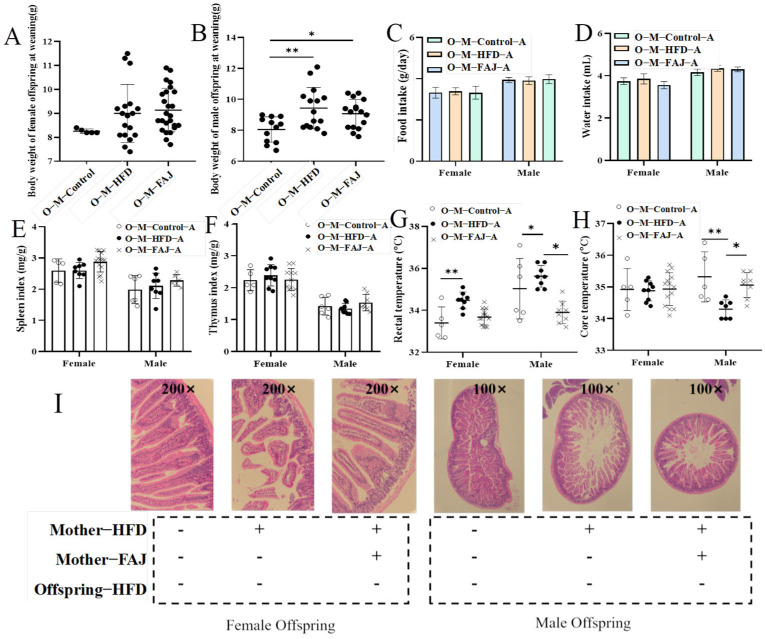
The weight of the offspring at weaning (**A**,**B**), feed intake (**C**), water intake (**D**), spleen index (**E**), thymus index (**F**), rectal temperature (**G**), core temperature (**H**), and jejunum injury (**I**) of allergic offspring mice from mothers with different diets. Each value is expressed as the mean ± SD. * *p* < 0.05 and ** *p* < 0.01.

By recording the body weights of weaned mice, it was found that maternal HFD increased the weight of the male offspring at weaning (*p* = 0.0049) but had no significant effect on the female offspring (Figure 3A,B). Hawkins et al. [25] reported a significant association between maternal pre-pregnancy overweight status and offspring adiposity, with children of overweight mothers exhibiting 1.37-fold higher odds of being overweight at 3 years compared to children of normal-weight mothers. In addition, there are strong positive associations between high birth weight and childhood overweight [26]. To investigate the effects of maternal HFD on the response of offspring mice to FA, the offspring mice in the M-Control, M-HFD, and M-FAJ groups were sensitized to OVA separately. It was found that food intake, water intake, spleen index, and thymus index (Figure 3C–F) in all groups were not observed to be different in offspring mice. However, maternal HFD resulted in a significantly higher rectal temperature in both male and female offspring as well as a lower core temperature in male offspring compared to the control group (*p* = 0.0466). However, FAJ intervention in mothers with HFD induced a marked decrease in rectal temperature and an increase in core temperature (Figure 3G,H). FA affects body temperature in mice by decreasing blood supply temperature due to vasodilation. Bai et al. [27] demonstrated that maternal HFD during pregnancy and lactation is associated with increased susceptibility to experimental FA in offspring mice. It is also important to emphasize that maternal obesity significantly potentiates offspring susceptibility to cow’s milk protein-induced food allergy in Balb/c mice [28].

### 3.4. Histological Evaluation of Offspring Mice

Intestinal villi serve as a selective barrier against pathogens and allergens, with their structural integrity directly correlating with intestinal functional capacity [29]. Histopathological assessment of the jejunum is shown in Figure 3I. The allergic offspring mice from the M-HFD group had more significant intestinal damage compared to those from the M-Control group. Specifically, the arrangement of jejunal wool was disordered, with an irregular shape, and the spacing of epithelial cells was widened. Nevertheless, allergic offspring mice from the M-FAJ group demonstrated less intestine damage, indicating that the FAJ intervention not only reduced anaphylaxis caused by OVA but also decreased intestine damage caused by allergic symptoms. Consistent with our study, Gao et al. [28] demonstrate that maternal exposure to HFD during pregnancy and lactation impaired intestinal epithelial integrity of the small intestine in offspring mice.

### 3.5. Serological Analysis of Offspring Mice

**Figure 4:**
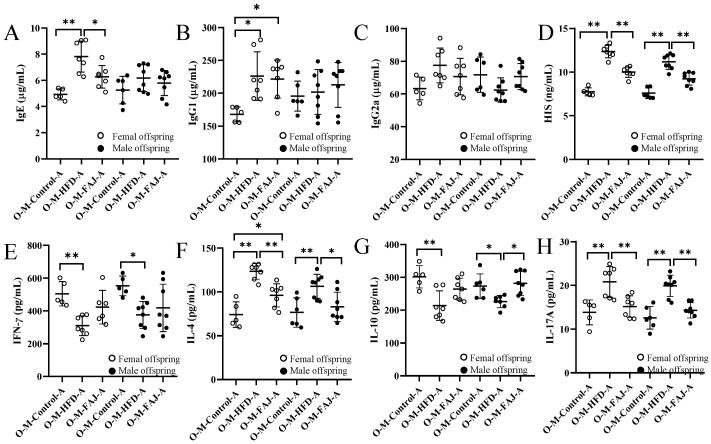
Content of IgE (**A**), IgG1 (**B**), IgG2a (**C**), HIS (**D**), IFN-*γ* (**E**), IL-4 (**F**), IL-10 (**G**), and IL-17A (**H**) in allergic offspring from mothers with different diets. Each value is expressed as the mean ± SD. * *p* < 0.05 and ** *p* < 0.01.

Next, we analyzed serological indicators, including IgE, IgG1, IgG2a, HIS, and cytokines representing Th1 (IFN-γ), Th2 (IL-4), Treg (IL-10), and Th17 (IL-17A) immune responses. Although male offspring in the O-M-HFD-A and O-M-Control-A groups showed no significant differences in IgE, IgG1, and IgG2a levels, female offspring in the O-M-HFD-A group exhibited elevated IgE, IgG1, and HIS levels compared to the O-M-Control-A group (Figure 4A–D). This suggests that maternal HFD exacerbates FA in offspring mice, consistent with findings by Xue et al. [7] that maternal obesity promotes inflammation in offspring mice. FAJ intervention to mothers resulted in the decrease of IgE and HIS contents by 19.91% and 19.00%, respectively, compared to the female offspring mice in the O-M-HFD-A group. Similarly, the representative cytokines IL-4, IFN-*γ*, IL-10, and IL-17A were measured to explore the effect of maternal HFD on Th1/Th2 and Treg/Th17 balances of offspring mice. As demonstrated in Figure 4E–H, maternal HFD significantly increased the levels of IL-4 and IL-17A in mice while reducing the content of IFN-*γ* and IL-10 (*p* < 0.05) in offspring. These findings suggest that maternal HFD during pregnancy and lactation may cause more serious allergic reactions. Nevertheless, the intervention of FAJ in mothers alleviated the OVA-induced Th1/Th2 and Treg/Th17 imbalance in offspring, which was similar to the results of Ma et al. [18] Gao et al. [28] also indicated that maternal HFD altered systemic and local inflammation response in offspring mice.

### 3.6. Gut Microbiota of Offspring Mice

**Figure 5:**
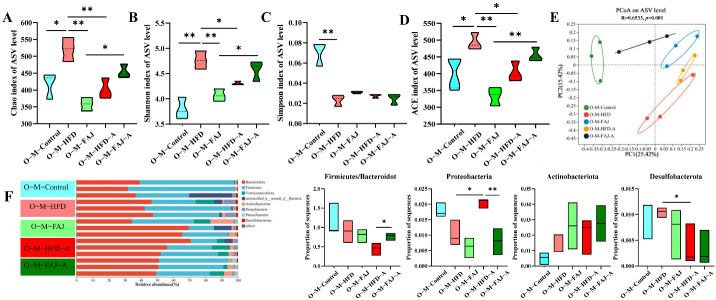
Effect of a maternal HFD during pregnancy and lactation on the richness and diversity of gut microbiota in offspring mice. (**A**) Chao index. (**B**) Shannon index. (**C**) Simpson index. (**D**) ACE index. (**E**) PCoA scores plot of gut microbiota at the ASV level. (**F**) The composition of gut microbiota at the phylum level. Each value is expressed as the mean ± SD (*n* = 3). * *p* < 0.05 and ** *p* < 0.01.

To further investigate the impact of maternal dietary habits during pregnancy and lactation on offspring gut microbiota, we selected female offspring mice for analysis. Gut microbiota diversity was characterized by Shannon, Simpson, ACE, and Chao1 indices. It is worth noting that maternal HFD during pregnancy and lactation significantly increased the alpha diversity of offspring gut microbiota, while FAJ intervention decreased the Chao, Shannon, and ACE indexes (*p* < 0.05) (Figure 5A–D). Our findings demonstrate that maternal HFD enhanced microbial diversity and richness in offspring, while FAJ supplementation attenuated these HFD-induced effects, ultimately decreasing alpha diversity. However, the alpha diversity increased in the O-M-FAJ-A group and decreased compared to pre-sensitization (O-M-FAJ) (*p* < 0.05), indicating that FAJ intervention to mothers had the potential to protect offspring mice from the allergic effects on gut microbiota. PCoA at ASV levels showed that the composition of the gut microbiota in the O-M-FAJ-O group was closer to the O-M-Control group (Figure 5E), which means FAJ supplementation significantly increased microbial *α*-diversity. Ma et al. [30] found that consuming HFD during pregnancy persistently altered the gut microbiota structure of the offspring. A population-based cohort study indicated that maternal HFD during pregnancy and lactation leads to significant changes in the gut microbiota of newborns from birth to 4–6 weeks of age [31].

**Figure 6:**
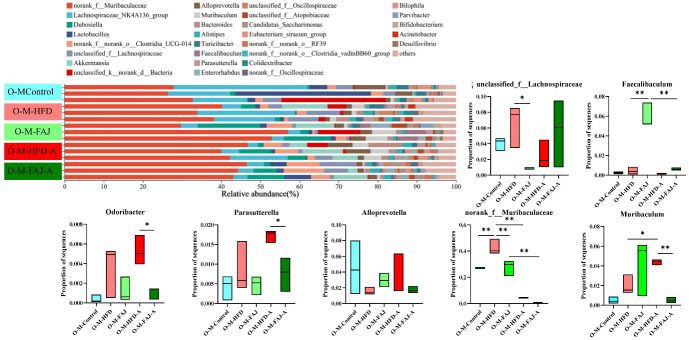
The composition of gut microbiota at the genus level. Each value is expressed as the mean ± SD (*n* = 3). * *p* < 0.05 and ** *p* < 0.01.

At the phylum level, the different maternal dietary habits did not significantly affect the relative abundance of the offspring’s gut microbiota in terms of the ratio of Firmicutes/Bacteroidota, Actinobacteriota, Desulfobacterota, and Proteobacteria (*p* > 0.05). Sensitization by OVA significantly down-regulated the percentage of Desulfobacterota and up-regulated the abundance of Proteobacteria in the offspring mice (*p* < 0.05), while the relative abundance of Proteobacteria in the O-M-FAJ-A group significantly increased. This is consistent with the conclusion of Ma et al. [32]. In the O-M-HFD-A group, the ratio of Firmicutes/Bacteroidota was reduced by 50.43% (*p* > 0.05) compared to the O-M-HFD group, whereas the ratio significantly rebounded in the O-M-FAJ-A group (Figure 5F). At the genus level, *norank_f__Muribaculaceae*, *Lachnospiraceae_NK4A136_group*, *Dubosiella, Lactobacillus*, *norank_f__norank_o__Clostridia_UCG-014*, and *unclassified_f__Lachnospiraceae* were the main constituent flora. *Unclassified__f__Lachnospiraceae*, *Faecalibaculum*, *Odoribacter*, *Parasutterella*, *Alloprevotella*, *norank__f__Muribaculaceae*, and *Muribaculum* were all affected to varying degrees by different diets of the maternal generation during pregnancy and lactation. Comparison of the O-M-HFD-A and O-M-FAJ-A groups demonstrates that maternal FAJ intervention significantly affected changes in *Odoribacter*, *Parasutterella*, and *Muribaculum* in offspring-sensitized mice (*p* < 0.05) (Figure 6). In summary, maternal HFD during pregnancy and lactation provides the offspring mice with a richer and more diverse gut microbiota through the vagina, placenta, amniotic fluid, and breast milk [33]. However, FAs are influenced by multiple factors. When a FA occurs in offspring mice due to other external conditions, maternal HFD during pregnancy and lactation exacerbates the severity of the allergic symptoms. Fortunately, interventions with FAJ could help alleviate FA.

**Figure 7:**
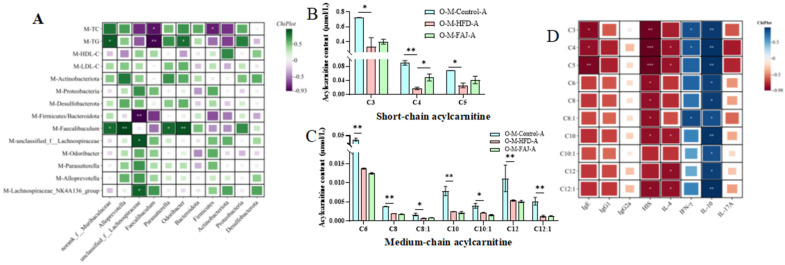
Correlation analysis between gut microbiota and lipid level of mothers and gut microbiota of offspring (**A**). Content of short-chain acylcarnitine (**B**) and medium-chain acylcarnitine (**C**) in serum of allergic offspring mice. Correlation analysis between cytokines and acylcarnitine of offspring (**D**). Each value is expressed as the mean ± SD. * *p* < 0.05, ** *p* < 0.01, and *** *p* < 0.001.

Lack’s dietary fat hypothesis [6] posits that increased dietary fat consumption contributes to the rising prevalence of both FA and metabolic disorders, including in pregnant populations. Therefore, this study proposes a scientific hypothesis that changes in maternal lipid levels may increase the risk of FA in offspring. Previous research has demonstrated that dyslipidemia may contribute to increased allergy risk [34]. The intricate relationship between gut microbiota and lipid levels of mothers and offspring’s allergic symptoms prompted us to investigate whether FA risk in offspring mice is related to maternal eating habits during pregnancy and lactation. Correlation analysis revealed that maternal HFD during pregnancy and lactation induced gut dysbiosis and lipid metabolic changes, characterized by increases in TG and TC and expansion of the phylum Feacalibaculum (Figure 7A), which was strongly associated with a high FA risk in offspring mice. These effects may be mediated through the vagina, placenta, amniotic fluid, breast milk, etc. [33]. It has been proved that gut microbiota may be transferred from the mother to the infant’s gut for colonization, thereby affecting infant gut microbiota immune system development [35]. Meanwhile, TGs with longer carbon chains and more double bonds were significantly associated with lower FA risk in offspring [36]. The above results indicated that maternal dietary conditions have a long-term effect on the offspring. Ma et al. [32] suggested that FAJ could alleviate FA through immune homeostasis and modulation of gut microbes. We speculated that FAJ has a positive effect on the gut microbiota of the mothers. This effect may be transmitted to the offspring through maternal pregnancy and breastfeeding, providing protective benefits when the offspring face FA. However, the specific mechanisms still require further investigation.

### 3.7. Acylcarnitine Levels in Offspring Mice

Biologically, carnitine exists in either its free (non-esterified) form or as esterified derivatives collectively referred to as acylcarnitines (AC) [37]. AC is produced in mitochondria as part of fatty acid metabolism and has been linked to metabolic disorders, cardiovascular disease, diabetes, depression, neurological disorders, etc. It has been shown that abnormal levels of carnitine in serum may affect metabolic disorders in offspring [38]. The above studies found that maternal HFD during pregnancy and lactation may lead to an increased risk of FA in the offspring mice, and thus the present study hypothesized that AC levels may be a potential marker of high FA risk in the offspring. A further study examined the levels of more than 40 AC of different carbon chain lengths in the serum of allergic offspring mice.

As depicted in Figure 7B,C, there was a significant reduction in the levels of three short-chain AC, namely propionylcarnitine (C3), butyrylcarnitine (C4), and isovalerylcarnitine (C5), along with seven medium-chain AC, including hexanoylcarnitine (C6), octanoylcarnitine (C8), octenoylcarnitine (C8:1), decanoylcarnitine (C10), decenoylcarnitine (C10:1), laurylcarnitine (C12), and lauroenoyl carnitine (C12:1), in the O-M-FAJ-A group (*p* < 0.05). Notably, the level of butyrylcarnitine (C4) in the O-M-FAJ-A group rebounded significantly (*p* < 0.05), while other short- and medium-chain AC exhibited different, but non-significant, trends towards rebounding. AC, as ester compounds formed by the combination of carnitine and fatty acids, reflect the metabolic capacity of fatty acids and organic acids of different chain lengths [39]. Short-chain AC plays roles in cellular energy metabolism by exerting antioxidant and anti-oxidative stress, regulating the peripheral nervous system function, and promoting nerve regeneration effects [40]. The anthropological analysis conducted by Pickens and Petritis [41] underscores that early nutritional deficiencies in newborns directly impact their physical and intellectual development, while nutrient excess also increases the risk of metabolic disorders in adulthood. In this study, the association between high FA risk and changes in certain levels of short-chain and medium-chain AC is noted. Short-chain AC was negatively correlated with IgE, HIS, and IL-4 levels and positively correlated with IFN-*γ* and IL-10 levels. Medium-chain AC was negatively correlated with HIS level and positively correlated with IL-10 level; C10, C12, and C12:1 were negatively correlated with IL-4 level, and C8:1 was positively correlated with IFN-*γ* level (Figure 7D). During the critical periods of pregnancy and lactation, HFD is an unavoidable process for the health of the mother and the development of the offspring. Our results indicated that maternal HFD during pregnancy and lactation increased the risk of FA in the offspring, and the levels of partial short- and medium-chain AC in the serum of the offspring may serve as potential biomarkers, which provided clinical recommendations for the early prevention and intervention of FA.

This study highlighted the relationship between maternal blood lipid levels, gut microbiota, and offspring gut microbiota. Changes in the gut microbiota played a key role in this process. Meanwhile, the level of AC in serum may serve as a biomarker related to FA. However, the mechanism by which the gut microbiota affects FA requires further research.

## 4. Conclusions

Maternal HFD during pregnancy and lactation exacerbated FA symptoms in offspring by regulating immune balance, destroying the intestinal barrier, and decreasing the diversity of intestinal microorganisms, which was confirmed. The intervention of FAJ during pregnancy and lactation showed promise in alleviating FA symptoms in offspring. Meanwhile, correlation analysis indicated that the maternal blood lipid level and gut microbiota were closely related to the gut microbiota of the offspring. Furthermore, the AC levels in offspring may serve as a potential marker for assessing the impact of maternal dietary habits on the risk of FA in offspring; the marker effect of AC levels on FA still needs to be further verified in allergic populations.

## Data Availability

The data presented in this study are available on request from the corresponding authors.

## Supplementary Materials

The following supporting information can be downloaded at https://www.mdpi.com/article/10.3390/nu17111927/s1: Figure S1: PCoA analysis in Lactobacillus plantarum PA01 and apple juice with different treatments (A). And the volcanic map of metabolites in 200 MPa+fermentation vs 200 MPa (B) and 200 MPa+fermentation vs pasteurization+fermentation (C). Red and blue dots represented an up-regulated and down-regulated expression of differential metabolites, respectively, and gray dots represented non-significant differential substances in groups. Differential metabolites in different groups (D). Untreated: apple juice without treatment; PA01: Lactobacillus plantarum PA01; 200 MPa: apple juice treated with 200 MPa; 200 MPa+Fermentation: apple juice treated with 200 MPa and fermented with Lactobacillus plantarum PA01; Pasteurization+Fermentation: pasteurized apple juice and then fermented with Lactobacillus plantarum PA01.
