# Supplementary material for: Fermented Apple Juice Reduces the Susceptibility of Offspring Mice to Food Allergy Exacerbated by Maternal High-Fat Diet
Source: Nutrients. 2025 Jun 4;17(11):1927. doi: 10.3390/nu17111927 (PMC12157162; doi:10.3390/nu17111927)
Supplement: Supplementary file 1 [file nutrients-17-01927-s001.zip › nutrients-3668697-supplementary.pdf]

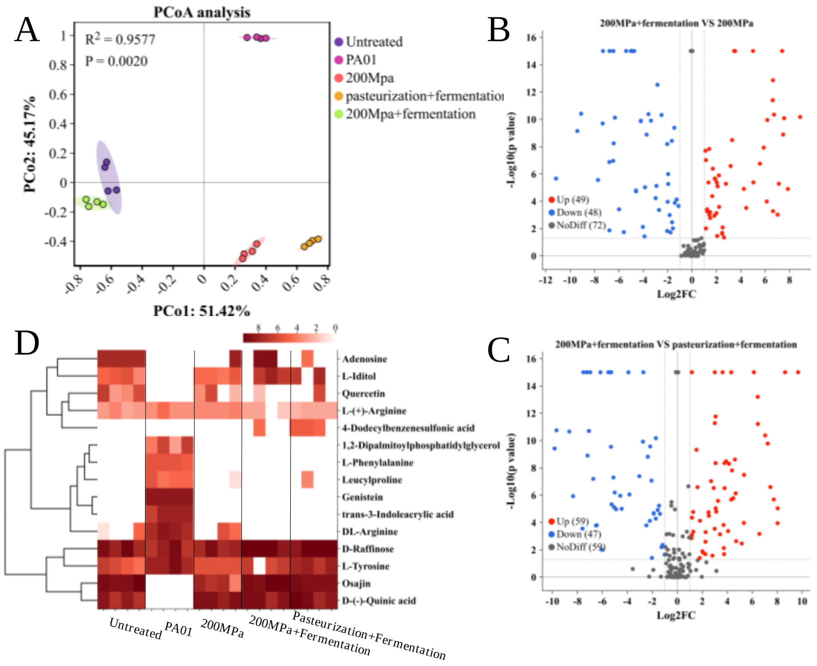

Fig. S1 PCoA analysis in *Lactobacillus plantarum PA01* and apple juice with different treatments (A). And the volcanic map of metabolites in 200 MPa+fermentation vs 200 MPa (B) and 200 MPa+fermentation vs pasteurization+fermentation (C). Red and blue dots represented an up-regulated and down-regulated expression of differential metabolites, respectively, and gray dots represented non-significant differential substances in groups. Differential metabolites in different groups (D). Untreated: apple juice without treatment; PA01: *Lactobacillus plantarum PA01*; 200 MPa: apple juice treated with 200 MPa; 200 MPa+Fermentation: apple juice treated with 200 MPa and fermented with *Lactobacillus plantarum PA01*; Pasteurization+Fermentation: pasteurized apple juice and then fermented with *Lactobacillus plantarum PA01*.
